# The Intergenerational Effects on Birth Weight and Its Relations to Maternal Conditions, São Paulo, Brazil

**DOI:** 10.1155/2015/615034

**Published:** 2015-02-01

**Authors:** Leide Irislayne Macena da Costa e Silva, Filumena Maria da Silva Gomes, Maria Helena Valente, Ana Maria de Ulhôa Escobar, Alexandra Valéria Maria Brentani, Sandra J. F. E. Grisi

**Affiliations:** ^1^Pediatrics Department, Medical School (FMUSP), University of São Paulo (USP), 05508-070 São Paulo, SP, Brazil; ^2^Hospital Universitário (HU), University of São Paulo (USP), 05508-000 São Paulo, SP, Brazil

## Abstract

*Background and Objectives.* Parents' birth weight acts as a predictor for the descendant birth weight, with the correlation more strongly transmitted through maternal line. The present research aims to study the correlation between the child's low or increased birth weight, the mother's birth weight, and maternal conditions.* Methods.* 773 mother-infant binomials were identified with information on both the baby's and the mother's birth weight recorded. Group studies were constituted, dividing the sample according to birth weight (<2,500 grams (g) and ≥3,500 grams (g)). The length at birth was also studied in children ≤47.5 cm (lower quartile). Chi^2^ test or Fisher's exact test, Spearman's Rho, and odds ratio were performed in order to investigate the relation between the children's weight and length at birth and the mothers' and children's variables.* Results.* The girls were heavier at birth than their mothers, with an average increase at birth weight between the generations of 79 g. The child's birth weight <2,500 g did not show any correlation with maternal birth weight <2,500 g (*Fisher 0.264; Spearman's Rho 0.048; OR 2.1 and OR lower 0.7*) or with maternal stature below the lower quartile (<157 cm) (*Chi* ^*2*^ *sig 0.323; with Spearman's Rho 0.036; OR 1.5 and OR lower 0.7*). The child's low birth weight (<2,500 g) was lightly correlated with drug use by the mother during pregnancy (*Fisher 0.083; Spearman's Rho 0.080; OR 4.9 and OR lower 1.0*). The child's birth weight <2,500 g showed increased correlation with gestational age lower than 38 weeks and 3 days (*Chi* ^*2*^ *sig 0.002; Spearman's Rho 0.113; OR 3.2 and OR lower 1.5*). The child's weight at birth ≥3,500 g showed strong correlation with maternal weight at birth ≥3,500 g (*Chi* ^*2*^ *sig 0; Spearman's Rho +0.142; OR 0.5 and OR upper 0.7*). It was also revealed that the higher the maternal prepregnancy BMI, the stronger the correlation with child's birth weight ≥3,500 g ((maternal prepregnancy BMI > 25.0 with* Chi* ^*2*^ *sig 0.013; Spearman's Rho 0.09; OR 1.54 and OR upper 2.17*) and (maternal prepregnancy BMI > 30.0 with* Chi* ^*2*^ *sig 0 Spearman's Rho 0.137; OR 2.58 and OR upper 4.26*)). The child's length at birth in the lower quartile (≤47.5 cm) showed strong correlation with drug use by the mother during pregnancy (*Chi* ^*2*^ *sig 0.004; Spearman's Rho 0.105; OR 4.3 and OR lower 1.5*). *Conclusions.* The mother's increased weight at birth and the prenatal overweight or obesity were correlated with increased weight and length at birth of the newborn, coupled with the tendency of increasing birth weight between generations of mothers and daughters. Also, descendants with smaller length at birth are the children of women with the lowest statures.

## 1. Introduction

Intergenerational effect on birth weight studies has shown that the offspring's birth weight is related to the birth weight of both parents, with the correlation being more strongly transmitted through the maternal line [1, 2]. Women born with low birth weight have a higher risk of also having low birth weight children [3, 4]. Recently it has been proven that being born large for gestational age (LGA) is strongly correlated with maternal increased birth weight and also with mother's prepregnancy overweight or obesity [5, 6].

Fetal growth is a critical part of the prenatal period and can affect the health of the child both in short term and in long term. Small or large newborns face an increased risk of infant mortality and a variety of latter health problems, including metabolic and cardiovascular diseases in adulthood [7].

The possible mechanisms that explain the intergenerational effects on birth weight and linear growth are not mutually exclusives and include mechanical factors caused by reduced space for the fetus to grow, fetal programming of metabolic alterations, shared genetic characteristics, epigenetic effect, and sociocultural factors [8].

One of the main determinants of birth weight is maternal height. Maternal height is correlated to the mother's own birth weight [9].

The intergenerational cycle of growth failure is well known in developing countries. Children who suffered from malnutrition during gestational life and early childhood tend to have a shorter stature in adulthood and are more likely to have children with low birth weight. When these children who are born small are girls, they probably tend to perpetuate the malnutrition cycle [10].

Adverse exposures during preconception and pregnancy periods (such as work load, preexisting maternal illnesses, pharmacological treatments, exposure to pollutants, and imbalance between intake and energy expenditure) may also influence the duration of gestation period and the newborn's birth weight. This leads to an intergenerational transmission of altered birth weight [11].

The intergenerational transmission of low or increased birth weight and its delayed effects later in life are a matter of concern throughout the nations. Women who were born small for gestational age have an increased risk of developing hypertension during pregnancy, which may lead to giving birth to low birth weight newborns. This leads to an inherited predisposition to low birth weight and to cardiovascular risk [12]. Women with obesity and/or preexisting diabetes mellitus and/or gestational diabetes tend to give birth to children with increased birth weight, increased ponderal index, and increased future risk of obesity and diabetes mellitus in adulthood and also during their future pregnancies [13].

Maternal prepregnancy BMI (body mass index) is influenced by her own growth and development during intrauterine life and childhood. Maternal intrauterine life and childhood growth also have an impact on maternal stature, fat-free mass, placenta size, uterus and ovaries size, and maternal metabolism. All these variables affect female reproductive quality [8, 14].

This paper analyzes the intergenerational transmission of birth weight in two successive generations. The correlation between altered birth weight with the mother's birth weight and other maternal conditions was studied in full-term newborns in the “Hospital Universitário” of the University of São Paulo.

## 2. Methods

Mother-child binomials were only included as participants in this study if their birth weight was registered in their respective medical records. The following participants were excluded from this research: mothers who have given birth at home, subjects born from multiple gestation pregnancy, and also those who had congenital disorders. Stillborn babies were also excluded. Premature newborns (gestational age shorter than 37 weeks) were excluded due to the multicausal origins involved in preterm birth. Furthermore, the previously mentioned intergenerational effect is not consistent among preterm births [4].

The participants of this research were women who gave birth in the “Hospital Universitário” (HU) between January 2012 and March 2014. If the mother was also born in HU, their medical record was obtained using their birth date, full name, and their own mother's hospital record. Maternal birth weight was obtained either through the medical records found in HU or through the medical records located in the two biggest maternity hospitals from the area of HU.

558 mother-child binomials were formed. If the children born between January 2012 and March 2014 were not their mothers' first child, their older siblings were also included in this study in case they were also born in HU. Therefore, 773 mother-child binomials were obtained from the initial 558 women who gave birth between January 2012 and March 2014 in HU.

For analytical purposes, the collected data was analyzed according to three different parameters. For each one of these parameters, a group with two categories was formed. Firstly, the sample was sorted according to the criteria of low birth weight (Group I): those born with a birth weight lower than 2,500 grams (g) formed one category, while the rest formed another. Medical literature describes low birth weight as any weight <2,500 g [15]. In another moment, the sample was yet again sorted according to the criteria of increased birth weight (Group II): those born heavy (≥3,500 g) formed one category, while the other infants formed another. Kumar et al. [16] showed that full-term newborns with birth weight ≥3,500 g are above the 97th percentile. Lastly, the sample was sorted according to newborns' length at birth (Group III): those born in the lower quartile (≤47.5 centimeters (cm)) were compared with the rest of the participants.

Sociodemographic data, maternal anthropometry during pregnancy, and gestational history were obtained through the medical records. Other information about gestational risk factors and the delivery was also obtained that way.

The gestational age (GA) was calculated using the* Capurro* method. The sample was sorted according to the gestational age and the newborns were placed in one of these three categories: preterm pregnancy (GA <37 weeks), full-term pregnancy (between 37 weeks and 41 weeks and 6 days), and postterm pregnancy (GA >42 weeks) [15, 17].

The women were sorted according to their ages in three different groups: (a) those who were 19 years old or younger, (b) those who were between 20 and 35 years old, and (c) those who were older than 35 years old [18–20]. They were also sorted according to their level of education: (a) those who had not finished elementary school or/and middle school, (b) those who had finished middle school, (c) those who had finished high school, (d) those who had not finished college, and (e) those who had finished college education.

They were also sorted according to parity: they were sorted into different categories if they had previously already given birth to other children or not and, if so, how many. They were sorted according to their history of prenatal care as well: those who did not receive any prenatal care formed one group, while the ones who received prenatal care formed another. Smoking, drinking, and drug use during pregnancy were also taken into consideration. Pregnancy related diseases (such as arterial hypertension, gestational diabetes mellitus, urinary tract infection, and others) were also assessed.

Maternal stature during pregnancy was also obtained. The women in the lower quartile (<157 cm) were compared to the ones who were taller than 157 cm.

The participants were also sorted according to their prepregnancy body mass index (BMI). They were divided in the following categories: BMI <18.5 Kg/m^2^ (low weight); BMI between 18.5 and 24.9 Kg/m^2^ (normal); BMI between 25.0 and 29.9 Kg/m^2^ (overweight); and BMI ≥30.0 Kg/m^2^ (obesity) [21, 22].

The weight gain during pregnancy was estimated from prepregnancy or early prenatal nutritional statuses. Adequate weight gain during pregnancy varies according to maternal prepregnancy weight. Weight gain should be (a) between 12.5 and 18.0 kg in women with low prepregnancy weight; (b) between 11.5 and 16.0 kg in women with adequate prepregnancy weight; (c) between 7.0 and 11.5 kg in overweight women; and (d) between 5.0 and 9.0 kg in obese women [23].

We also compared female newborns with male newborns. The newborns whose birth length was in the lower quartile (≤47.5 cm) were compared to the others. Parturition was classified as being either vaginal or by surgical cesarean section.

Descriptive statistical analyses of the parametrical variables were performed by calculating the average, standard deviation, and standard error. The results were expressed according to these parameters. In order to test the homogeneity of the groups in relation to their proportions,* Pearson's Chi-square test *or* Fisher's exact test* were performed [24, 25]. In order to measure the monotonicity of the curve,* Spearman's Rho* test and the analysis of odds ratio were performed [26].

This Research Project was approved by the Research Ethics Committee from the “Hospital Universitário” of the University of São Paulo.

## 3. Results

94.0% of the mothers were born in the city of São Paulo, 83.8% of which were born in “Hospital Universitário.” Average maternal birth weight was 3,110 g, with a standard deviation (SD) of 463 g. Information about maternal gestational age was only available for 29% of the women who participated in this research, of which 95.1% were full-term newborns, 8.0% had low birth weight (<2,500 g), and 18.6% had increased birth weight (≥3,500 g). Maternal age at birth varied from 12 to 40 years old and 29% of the women were 19 years old or younger at birth. 40.3% of them had finished high school (at least 11 years of formal education) by the time of the delivery and 2.0% of them had been to college.

15.2% of participants smoked during pregnancy and 3.0% of them also consumed alcohol and/or other drugs during pregnancy. 58.7% of the 773 mother-child binomials were primiparous and the abortion prevalence reached 14.0%.

9 mothers (*n* = 763) did not receive prenatal care. 28.9% had urinary tract infection during pregnancy, 21.1% had leukorrhea during pregnancy, and 7.2% had other kinds of infectious diseases (*n* = 762). 14.7% had other kinds of diseases, such as asthma, bronchitis, and allergic rhinitis (*n* = 764).

29.4% of the mothers (*n* = 752) were shorter than 157 cm (lower quartile) and 21.7% were taller than 166 cm (upper quartile). Average prepregnancy BMI (*n* = 628) was 24.1 Kg/m^2^ with a standard deviation of 4.5% Kg/m^2^. 5.3% of the mothers had a BMI <18.5 Kg/m^2^, 59.4% had a BMI between 18.5 Kg/m^2^ and 24.9 Kg/m^2^, and 24.4% of them had a BMI between 25.9 Kg/m^2^ and 29.9 Kg/m^2^. 11% of them had a BMI ≥30 Kg/m^2^. The weight gain during pregnancy (*n* = 610) was inadequate in 69.0% of them: 39.6% showed insufficient weight gain and 29.4% of them gained excessive weight during pregnancy.

51.6% of the newborns were female. 3.5% had low birth weight (<2,500 g), 70.0% weighted between 2,500 g and 3,500 g, and 26.5% had an increased birth weight (≥3,500 g). Average birth weight was 3,242 g, with a standard deviation (SD) of 421 g.

Table 1 shows the analysis of low birth weight children (<2,500 g) in comparison to the others. Low birth weight had a strong correlation with length at birth in the lower quartile (≤47.5 cm), with* Fisher *0^*^ *; Spearman's Rho 0.287; OR 28.6 and OR lower 8.5*. Low birth weight had also a significant correlation with shorter full-term gestational age. If the child is born 10 to 14 days after completion of 37 weeks of gestational age, he/she will be born with a significantly higher birth weight (*Chi* ^*2*^ *sig 0.002; Spearman's Rho 0.113; OR 3.2 and OR lower 1.5*).

Low birth weight (<2,500) had a slight correlation with noncommunicable diseases, such as asthma, bronchitis, and allergic rhinitis (*Chi* ^*2*^ *sig 0.025; Spearman's Rho 0.081; OR 2.6 and OR lower 1.1*), and also with drug use during pregnancy (*Fisher *0.083^*^ *; Spearman's Rho 0.080; OR 4.9 and OR lower *1.0).

Table 2 shows the analysis of increased birth weight children (≥3,500 g) in comparison to the others. Increased birth weight had a strong correlation with maternal increased birth weight (*Chi* ^*2*^ *sig 0; Spearman's Rho 0.142; OR *0.5^**^ *and OR upper 0.7*) and surgical cesarean section (*Chi* ^*2*^ *sig 0; Spearman's Rho 0.132; OR *0.5^**^ *and OR upper 0.8*).

Increased birth weight (≥3,500 g) also had a strong correlation with inadequate maternal prepregnancy BMI: BMI < 18.5 Kg/m^2^ (*Fisher *0.068^*^ *; Spearman's Rho −0.069; OR *2.70^**^ *and OR upper 7.81*); BMI ≥ 25.0 Kg/m^2^ (*Chi* ^*2*^ *sig 0.013; Spearman's Rho 0.09; OR 1.54 and OR upper 2.17*); and BMI ≥ 30.0 (*Chi* ^*2*^ *sig 0; Spearman's Rho 0.137; OR 2.58 and OR upper 4.26*).

**Figure 1:**
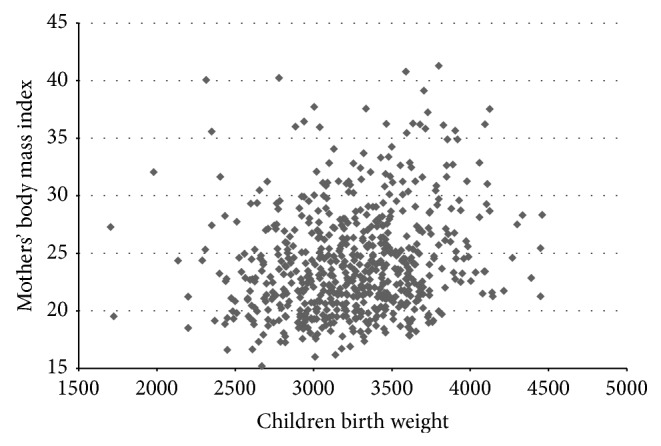
Correlation of maternal prepregnancy BMI and newborn birth weight.

Figure 1 shows a correlation between maternal prepregnancy body mass index (BMI) and newborn birth weight (*Spearman's Rho 0.210*). This correlation was more pronounced in newborns with birth weight >2,500 g.

Table 3 shows the analysis of children whose length at birth was in the lower quartile (≤47.5 cm) in comparison to the others. Length at birth in the lower quartile had a strong correlation with gestational age shorter than 38 weeks and 3 days (*Chi* ^*2*^ *sig 0; Spearman's Rho 0.134; OR 2.0 and OR lower 1.4*). Length at birth in the lower quartile also had a strong correlation with illicit drug use during pregnancy (*Chi* ^*2*^ *sig 0.004; Spearman's Rho 0.105; OR 4.3 and OR lower 1.5*).

Length at birth in the lower quartile (≤47.5 cm) had a slight correlation with maternal height in the lower quartile (<157 cm) (*Chi* ^*2*^ *sig 0.012; Spearman's Rho 0.091; OR 1.6 and OR lower 1.1*) and with smoking during pregnancy (*Chi* ^*2*^ *sig 0.012; Spearman's Rho 0.091; OR 1.7 and OR lower 1.1*).

## 4. Discussion

Similar researches conducted in Brazil analyzed data obtained through medical records. In the southern city of Pelotas, a cohort study followed 2.876 women born in 1982 through the years. 16% of them had at least one child [27]. Vélez et al. [28] conducted an intergenerational research which analyzed the medical records from 794 women of the Pelotas cohort. In this study, 25% of the data on the newborn birth weight was orally given by the mother and not by their medical records. It is important to highlight that it is common that such researches obtain information about maternal birth weight [29–31] and/or newborn birth weight [32] through maternal verbal report.

Systematized national databases are also consulted by researchers in developed countries that study the intergenerational transmission of birth weight. Emanuel et al. [33] and Hennessy and Alberman [34], for example, studied the birth weight from two generations of a British cohort in 1958. They followed the subjects until they were 23 [33] and 33 [34] years old, respectively. Recent Swedish investigations also consulted national medical records, which contained data on 98% of all the births which occurred in Sweden since 1973 and also data on both maternal and newborn birth weight [5, 6].

We were not able to retrieve data on the gestational age from 71.0% of the women who participated in this research. Literature shows, however, that maternal birth weight has a stronger correlation with newborn birth weight than maternal gestational age. Other studies have shown that mothers who were born small have a significantly increased risk of giving birth to a low birth weight child [29, 33, 35], but preterm mothers do not have an increased risk of having a preterm baby [29]. This suggests that the intergenerational cycle of growth failure may be linked to the fetus development and not to the gestation period duration [8, 33]. Alberman et al. [36] found a negative association between maternal gestational age and newborn birth weight. Magnus et al. did not find any correlation between maternal and infant gestational ages, which shows a lower heritability of gestational age across generations [3].

In this study, newborn average birth weight (3,242 g) was higher than maternal average birth weight (3,110 g). Both average birth weights were very similar to those found by Monteiro et al. in São Paulo, Brazil, [37] and also to those found by de Moraes et al.  (2012) in Rio Grande do Sul, Brazil [38].

Female newborns weighted on average 79 g more at birth than their mothers. Veena et al. also observed a birth weight increase between generations of women in southern India: in that study, the daughters' birth weight was on average 104 g heavier than their mothers' birth weight [1]. This tendency has also been observed in North American [39], European [40], Australian [41], and Chinese [42] populations.

Male newborns weighted on average 109 g more than female newborns. This result is very similar to the evidence given by Lubchenco et al. [43] and Alexander et al. [44]. This gender difference was also found by an Indian study, which revealed that boys are born on average 45 g heavier than girls [16].

Low birth weight (<2,500 g) was not correlated with low maternal birth weight. This differs from the results of other studies that showed an intergenerational transmission of low birth weight [3, 29, 33, 35, 45]. Vélez et al. found an association between low maternal birth weight with offspring's low birth weight and preterm birth. Maternal prepregnancy weight and young maternal age (<22 years) had an influence on the association of maternal low birth weight and newborn being small for gestational age. Teenage pregnancy is strongly related to adverse pregnancy outcomes [27, 28, 46].

Newborn low birth weight (<2,500 g) and newborn birth length in the lower quartile (≤47.5 cm) had a correlation with drug use by the mother during pregnancy. Newborn birth length in the lower quartile also had a slight correlation with smoking during pregnancy. Several studies in medical literature demonstrate that smoking and illicit drug use during pregnancy are related to newborn low birth weight [47–50]. de Stalova et al. have found that sociodemographic and behavioral factors also contribute moderately but significantly to the intergenerational transmission of low birth weight [2].

We did not find any correlation between newborn low birth weight (<2,500 g) and maternal height in the lower quartile (<157 cm). This differs from several studies that showed an increased risk of newborn low birth weight in women with short height when compared to reference height [51–55].

A recent Brazilian study that analyzed 2,226 mother-child binomials found that maternal height in the lower quartile (≤152 cm) was related to an increased risk (42%) for having children with low birth weight when compared to mothers in the upper quartile for height (>160.4 cm) [56]. However, the systematic review of Han et al. concludes that only studies with unadjusted data express associations between women of short stature and increased risk of premature birth or offspring with low birth weight [57].

Newborn increased birth weight (≥3,500 g) had a strong correlation with increased maternal birth weight. A similar result was observed in the study of Buffalo's birth cohort: Klebanoff et al. found that increased maternal birth weight was the best predictor for large newborns for gestational age after controlling for all confounding factors. Women who weighed more than 3,600 g at birth presented a higher risk of having a child large for their gestational age [58]. In the city of Porto (Portugal), Tavares et al. also found that increased birth weight was more common in children whose mothers were born weighting more than 4,000 g [59]. Ahlsson et al. [5] and Cnattingius et al. [6] strengthened this evidence by demonstrating that mothers who were born large for their gestational age showed an increased risk of having children equally large for their gestational age.

In the present study, newborn increased birth weight (≥3,500 g) also had a strong correlation with increased maternal prepregnancy BMI: the higher the mother's prepregnancy BMI was, the stronger the correlation with newborn increased birth weight was.

A meta-analysis has showed an association between prepregnancy overweight or obesity with increased risk for newborns large for gestational age (LGA) and babies with weight >4,000 g or macrosomic [60]. LGA babies have a higher risk of becoming overweight or obese adults [5]. This may lead to a “snowball” effect: LGA girls are more likely to become overweight or obese adults and thus have a higher risk of having babies also born large for gestational age [6].

Newborn birth length in the lower quartile (≤47.5 cm) had a slight correlation with maternal height in the lower quartile (<157 cm). Witter and Luke had a similar result: newborns whose mother's height varied from 150 to 157 cm were smaller than children born to taller women (between 168 cm and 175 cm) [61]. Veena et al. [1] also found that maternal height was positively associated with newborn birth length. In an Asian study with teenage mothers, newborn crown-heel length was also larger in maternal statures in the upper quartiles [62].

Medical literature shows that first-born children are usually lighter than their younger siblings [63–65]. 58.7% of the participants in this research were firstborns, a limitation that can be a bias in the observed tendency of correlation between increased maternal birth weight with newborn increased birth weight.

Studies have showed that maternal age is an important predictor for the size of the newborn: teenage mothers tend to have smaller babies [66]. 29.0% of the women who participated in our study were younger than 20 years old. However, this did not influence the result which showed a correlation between maternal and newborn increased birth weights. A similar finding was observed by Ahlsson et al. [5].

This research provides two evidences for the intergenerational vicious cycle of high birth weight: (1) increased maternal birth weight and prepregnancy maternal overweight or obesity had a strong correlation with newborn high birth weight; (2) a tendency of daughters having a higher birth weight than their mothers was observed.

Public policies may lead to important benefits for the next generations. Improving female health with a focus on preconception, maternal-fetal health, proper development of children, fight against obesity, and socioeconomic improvement may be a way of interruption of the intergenerational cycle of growth failure and the perpetuation of obesity in the world.

## 5. Conclusions

Newborn low birth weight was not correlated to low maternal birth weight. However, newborn high birth weight (≥ 3.500 g) had a strong correlation with high maternal birth weight.

Newborn low birth weight had a slight correlation with drug use during pregnancy. Increased birth weight had a strong correlation with increased maternal prepregnancy body mass index (BMI ≥ 25 mg/kg^2^), with absence of smoking during pregnancy, and also with surgical cesarean section.

Newborn birth length in the lower quartile (≤47.5 cm) had a strong correlation with drug use during pregnancy and a mild correlation with maternal stature in the lower quartile (<157 cm) and with smoking during pregnancy.

The results suggest that increased maternal birth weight (≥3.500 g) and maternal prepregnancy overweight or obesity is associated with newborn high birth weight. This points to a tendency of increasing weight across generations, which can result in an intergenerational cycle of obesity.

## Figures and Tables

**Table 1:** Low birth weight analysis (<2,500 g)—Group I.

Variable	Chi^2^	Spearman's Rho	OR	OR lower	OR upper
Maternal birth weight^1^ <2.500 g	0.264^*^	0.048	2.1	0.7	6.2
Maternal birth weight^1^ ≥3.500 g	0.126^*^	−0.058	2.6	0.8	8.6
Maternal age ≤19 years or ≥35 years	0.750^*^	0.006	1.1	0.3	3.7
Smoking during pregnancy^2^	0.114	0.057	2.0	0.8	4.9
Alcohol use during pregnancy^2^	1.000^*^	−0.007	1.2^**^	0.2	9.2
Illicit drug use during pregnancy^2^	0.083^*^	0.080	4.9	1.0	23.0
Arterial hypertension^2^	1.000^*^	0.001	1.0	0.2	4.5
Diabetes mellitus^2^	1.000^*^	−0.018	—	—	—
Urinary tract infections^2^	0.823	−0.008	1.1^**^	0.5	2.7
Leukorrhoea^2^	0.804	0.009	1.1	0.4	2.8
Others infectious diseases^2^	0.248^*^	−0.052	—	—	—
Anemia^2^	1.000^*^	−0.003	1.1^**^	0.1	8.4
Oligohydramnios^2^ or polyhydramnios^2^	0.555^*^	0.009	1.3	0.2	10.0
Noncommunicable diseases^2^	0.025	0.081	2.6	1.1	6.0
Maternal depression during pregnancy^2^	1.000^*^	−0.022	—	—	—
Maternal height <157 cm	0.323	0.036	1.5	0.7	3.3
Prepregnancy maternal BMI^3^ <18.5 kg/m^2^	1.000^*^	−0.005	1.17^**^	0.15	8.85
Prepregnancy maternal BMI^3^ ≥25 kg/m^2^	0.927	0.003	1.04	0.45	2.41
Prepregnancy maternal BMI^3^ ≥30 kg/m^2^	0.296^*^	0.038	1.79	0.6	5.34
Newborn height at birth ≤47.5 cm	0^*^	0.287	28.6	8.5	96.2
Gestational age (269–273 days)^4^	0.002	0.113	3.2	1.5	7.0
Surgical cesarean section	0.306	0.037	1.5	0.7	3.3

^*^Fisher test.

^**^With homogenization signal, if OR <1, then consider OR = 1/OR.

(—) Cell with zero occurrences, undefined OR.

^1^Maternal birth weight in grams.

^2^Maternal habits and diseases during pregnancy.

^3^Prepregnancy maternal BMI = prepregnancy body mass index maternal in Kg/m^2^.

^4^Gestational age in days (269–273) = gestational age varying from 38 weeks and 3 days to 39 weeks.

**Table 2:** Increased birth weight analysis (≥3,500 g)—Group II.

Variable	Chi^2^	Spearman's Rho	OR	OR lower	OR upper
Maternal birth weight^1^ <2.500 g	0.026	−0.08	0.4	0.2	0.9
Maternal birth weight^1^ ≥3.500 g	0	0.142	0.5^**^	0.3	0.7
Maternal age ≤19 years or ≥35 years	0.174	0.038	0.7^**^	0.4	1.2
Smoking during pregnancy^2^	0.011	−0.091	0.5	0.3	0.9
Alcohol use during pregnancy^2^	0.415	−0.029	0.7	0.3	1.6
Illicit drug use during pregnancy^2^	0.129^*^	−0.06	0.2	0.03	1.6
Arterial hypertension^2^	0.483	0.038	0.8^**^	0.4	1.5
Diabetes mellitus^2^	0.088^*^	0.066	0.3^**^	0.06	1.2
Urinary tract infections^2^	0.693	−0.014	0.9	0.7	1.3
Leukorrhoea^2^	0.082	0.063	0.7^**^	0.5	1.0
Others infectious diseases^2^	0.231	−0.043	0.7	0.3	1.3
Anemia^2^	0.572	0.021	0.8^**^	0.4	1.7
Oligohydramnios^2^ or polyhydramnios^2^	0.695	0.038	0.8^**^	0.3	2.1
Noncommunicable diseases^2^	0.253	0.041	0.8^**^	0.5	1.2
Maternal depression during pregnancy^2^	0.070^*^	−0.07	—	—	—
Maternal height <157 cm	0.406	−0.030	0.9	0.6	1.2
Prepregnancy maternal BMI^3^ <18.5 kg/m^2^	0.068^*^	−0.069	2.70^**^	0.94	7.81
Prepregnancy maternal BMI^3^ ≥25 kg/m^2^	0.013	0.09	1.54	1.01	2.17
Prepregnancy maternal BMI^3^ ≥30 kg/m^2^	0	0.137	2.58	1.56	4.26
Newborn height at birth ≤47.5 cm	0^*^	−0.305	0.05	0.02	0.1
Gestational age (269–273 days)^4^	0	−0.142	0.4	0.3	0.6
Surgical cesarean section	0	0.132	0.5^**^	0.4	0.8

^*^Fisher test.

^**^With homogenization signal, if OR <1, then consider OR = 1/OR.

(—) Cell with zero occurrences, undefined OR.

^1^Maternal birth weight in grams.

^2^Maternal habits and diseases during pregnancy.

^3^Prepregnancy maternal BMI = prepregnancy body mass index maternal in Kg/m^2^.

^4^Gestational age in days (269–273) = gestational age varying from 38 weeks and 3 days to 39 weeks.

**Table 3:** Length at birth in the lower quartile (≤47.5 cm)—Group III.

Variable	Chi^2^	Spearman's Rho	OR	OR lower	OR upper
Maternal birth weight^1^ <2.500 g	0.757	0. 011	1.1	0.6	2.0
Maternal birth weight^1^ ≥3.500 g	0	−0.154	3.2^**^	1.8	5.7
Maternal age ≤19 years or ≥35 years	0.175	0.049	1.4	0.9	2.4
Smoking during pregnancy^2^	0.012	0.091	1.7	1.1	2.6
Alcohol use during pregnancy^2^	0.619	−0.018	1.2^**^	0.5	2.9
Illicit drug use during pregnancy^2^	0.004	0.105	4.3	1.5	12.6
Arterial hypertension^2^	0.713	−0.013	1.1^**^	0.6	2.2
Diabetes mellitus^2^	0.673^*^	0.011	1.3	0.2	6.7
Urinary tract infections^2^	0.606	0.019	1.1	0.8	1.6
Leukorrhoea^2^	0.875	0.006	1.0	0.7	1.6
Others infectious diseases^2^	0.181	−0.048	1.6^**^	0.8	3.4
Anemia^2^	0.395^*^	−0.040	1.7^**^	0.7	4.5
Oligohydramnios^2^ or polyhydramnios^2^	0.205	0.046	1.7	0.7	4.2
Noncommunicable diseases^2^	0.425	0.029	1.2	0.8	1.9
Maternal depression during pregnancy^2^	0.262^*^	0.043	2.1	0.6	7.7
Maternal height <157 cm	0.012	0.091	1.6	1.1	2.2
Gestational age (269–273 days)^3^	0	0.134	2.0	1.4	2.9
Surgical cesarean section	0.672	−0.015	1.1^**^	0.8	1.6

^*^Fisher test.

^**^With homogenization signal, if OR <1, then consider OR = 1/OR.

(—) Cell with zero occurrences, undefined OR.

^1^Maternal birth weight in grams.

^2^Maternal habits and diseases during pregnancy.

^3^Gestational age in days (269–273) = gestational age varying from 38 weeks and 3 days to 39 weeks.
